# Modifying “Pico” Question into “Picos” Model for More Robust and Reproducible Presentation of the Methodology Employed in A Scientific Study

**Published:** 2017-09

**Authors:** Muhammad Saaiq, Bushra Ashraf

**Affiliations:** Pakistan Institute of Medical Sciences (PIMS), Shaheed Zulfiqar Ali Bhutto Medical University (SZABMU), Islamabad, 44000, Pakistan

**Keywords:** Pico, Picos, Presentation, Methodology, Study

**DEAR EDITOR**

The mnemonic “PICO” was originally coined to help guide a standardized and disciplined way of formulating a clinical research question, carrying out a thorough literature search to answer that question (particularly through the MEDLINE/PubMed) and resultantly generate a fulfilling and all encompassing evidence-based answer to the constructed clinical query. The elements of the PICO question included “P” for problem or patient or population, “I” for intervention or exposure, “C” for comparison and “O” for outcomes. The PICO question was recommended to be phrased into appropriate search strings to find out all the relevant published quality literature available on the cyberspace.^1^^-^^3^

The PICO framework is absolutely laudable for the purpose for which it was originally introduced. We modify it into PICOS model to further extend its scientific utility for the logical and thorough description of the methodology part of the scientific manuscripts. We include “S” for representing an equally important component of the research exercise. i.e. the statistical analyses employed for analyzing the data obtained from the study and the subsequent conclusions inferred there from. The advocated addition of “S” to the mnemonic PICO modifies it into PICOS, ensuring reproducibility and more robust expression of the study protocol followed in any particular scientific research.^1^^-^^3^

The crucial concept of statistical analyses in research studies is well established, however quite often researchers are unaware of its significance and logical implications. Resultantly their reported studies suffer the lack of robust reproducibility and translation to the general population at large.^4^^,^^5^ The proposed PICOS approach is intended to serve as a guide for the authors and help them efficiently and thoroughly describe their research methodology while reporting their original studies. Additionally it will also serve as a checklist guide for the reviewers and editors to more thoroughly review the manuscripts under their evaluation and hence ensure their scientific validity and statistical robustness. By ensuring uniform methodological standards and objectivity, the overall scientific value of the published literature will certainly enhance.^4^^,^^5^ Following is a brief summary of the proposed PICOS model for describing the methodology employed in any research pursuit: 

*P---Patient population: *The patients or subjects who were studied in the research pursuit are specifically highlighted. This essentially includes clear indication of the inclusion and exclusion criteria employed in recruiting the patients, the potential confounders controlled and the study design adopted. Mentioning these salient features ensures an exact definition of the population studied and helps eliminate any ambiguity in the interpretation of results. Also mentioning the hospital(s) or the setup where the study was undertaken and duration of the study ensures better understanding of the population under srutiny.^6^^,^^7^

*I---Intervention: *The intervention tested is described in clear understandable words. The intervention could be a new medicine (or a product for instance), some new surgical procedure (or a technique for that matter) or some other newer form of therapy. Brief elaboration of the relevant attributes of the intervention under scrutiny is ensured in the methodological details. This may include the dosage and regimens of the medications used or the salient features of the surgical approach followed.^6^^,^^7^

*C---Comparative controls:* The alternative treatment strategy or a normal group of individuals which was used as a comparative tool in the current study is clearly mentioned and appropriately elaborated. For instance the control group could have been treated with a previously established treatment protocol or subjected to a placebo or nocebo therapy. Brief elaboration of the method of randomization of the patients to either the intervention group or the controls is greatly desirable. Any blinding or masking employed to reduce bias are also mentioned. If a study has not recruited controls as part of its methodology (for example in a case series or descriptive study), it is prudent for authors to mention this shortcoming as the limitation of the study at an appropriate place in the discussion part of the manuscript. This helps to prompt the readers to interpret the results keeping in view this major methodological drawback of the study.^6^^,^^7^

*O---Outcome(s):* The outcome measure(s) of interest or the study’s target end points evaluated in the given research are objectively defined. There could be primary outcome measures only or additionally, there may be secondary outcome measures scrutinized as well. These should be logically mentioned accordingly. Objective narration of the measuring devices used, the target follow up periods and any drop-outs from the follow up appointments, greatly helps in correct interpretation of the results of the study.^6^^,^^7^

*S---Statistical analysis (analyses):* The different statistical methods and tests employed to analyze the data yielded by the research are precisely mentioned. The various statistical tools used to calculate the numerical and categorical data of the study are expressed accordingly. The statistical tests such as chi-square test applied and the significance level set are all mentioned. The statistical software such as the SPSS (Statistical package for social sciences) or any other similar software employed is mentioned. Statistically robust studies would prudently include sample size calculation and power analysis. This helps to determine the number of subjects needed in order to have a reasonable chance of showing a difference if it truly exists. A sufficiently powered study certainly has less chance of errors.^6^^,^^7^

By adopting the proposed PICOS framework (i.e. Patient population- Intervention- Comparative controls*- *Outcomes- Statistical analyses), it is possible to ensure scientific thoroughness and objectify reporting of the methodology part of any scientific manuscript. This will ensure reproducibility of the current study as well as its comparability to other similar studies carried out by other scientific colleagues working in other institutes in any other part of the world.^6^^,^^7^

## CONFLICT OF INTEREST

The authors declare no conflict of interest.
